# The Plasmid-Borne *tet*(A) Gene Is an Important Factor Causing Tigecycline Resistance in ST11 Carbapenem-Resistant *Klebsiella pneumoniae* Under Selective Pressure

**DOI:** 10.3389/fmicb.2021.644949

**Published:** 2021-02-24

**Authors:** Juan Xu, Zhongliang Zhu, Yanmin Chen, Weizhong Wang, Fang He

**Affiliations:** ^1^School of Public Health, Hangzhou Medical College, Hangzhou, China; ^2^Department of Clinical Laboratory, Zhejiang Provincial People’s Hospital, People’s Hospital of Hangzhou Medical College, Hangzhou, China

**Keywords:** mutation, plasmid-bearing, tigecycline, *tet*(A), *Klebsiella pneumoniae*

## Abstract

The emergence and prevalence of tigecycline-resistant *Klebsiella pneumoniae* have seriously compromised the effectiveness of antimicrobial agents in the treatment of infections. To explore the role of the plasmid-borne *tet*(A) gene in tigecycline resistance in carbapenem-resistant *K. pneumoniae* (CRKP), a total of 63 CRKP isolates were collected from a tertiary hospital in Hangzhou, China. The minimum inhibitory concentration (MIC) of tigecycline, mutation rate of *tet*(A) gene, genetic surroundings of *tet*(A)-carrying transmissible plasmid and the contribution of *tet*(A) mutation to tigecycline resistance were analyzed using antimicrobial susceptibility test, whole-genome sequencing, tigecycline resistance evolution experiment, and plasmid conjugation experiment. Our results showed that 52.4% (33 isolates) of the test isolates carried the *tet*(A) gene; among them, 75.8% (25 isolates) exhibited a tigecycline non-susceptible phenotype (MIC = 4 mg/L). Three clonal groups (cluster I, cluster II, and cluster III) were identified in these *tet*(A)-bearing isolates. All 17 isolates belonged to serotype KL21 (cluster I), which differed by only 13 SNPs, suggesting a clonal spread of *tet*(A)-positive ST11 *K. pneumoniae* with serotype KL21 occurred in the sampling hospital. The induction of tigecycline resistance experiments showed that 71.4% of strains evolved *tet*(A) mutations and developed a high-level tigecycline resistance. Eight amino acid substitutions were identified in these mutants. The most common amino acid substitution was A370V, followed by S251A and G300E. Twelve isolates carrying *tet*(A) mutants succeeded in the filter mating experiment with a conjugation efficiency of 10^–3^–10^–8^. Tigecycline MICs in *E. coli* EC600 transconjugants with a mutated *tet*(A) were 2 to 8-fold higher than those in *E. coli* EC600 transconjugants with a wild-type *tet*(A). One ColRNAI/IncFII type and two IncFII type *tet*(A)-bearing conjugative plasmids were identified in this study, including a class 1 integron containing multiple antibiotic resistance genes, i.e., *tet*(A), *qnrS1*, *bla*_*LAP–*__2_, *catA2*, *sul2*, and *dfrA14*. Our study revealed the wide-spread situation of plasmid-borne *tet*(A) gene in clinical CRKP, and mutation of *tet*(A) is a potential driven force that lead to tigecycline resistance.

## Introduction

Carbapenem-resistant *Klebsiella pneumoniae* (CRKP) is currently a substantial threat to public health worldwide. CRKP can cause a variety of infections, such as pneumonia, liver abscess, urinary tract infection, and bloodstream infection. CRKP often carry multiple antimicrobial resistance genes in the chromosome and plasmids, enabling the strain to be resistant to almost all antibiotics, except colistin and tigecycline. Tigecycline, the first glycylcycline drug, is an extended-spectrum antibiotic that inhibits protein synthesis by binding to the 30S ribosome and can overcome the mechanisms of tetracycline resistance (Pankey, 2005).

Antibiotics that can treat CRKP infections are limited. Tigecycline remains an important treatment method for CRKP. However, tigecycline resistance has emerged since the approval of this antibiotic and has been reported frequently in Enterobacteriaceae (Hoban et al., 2005). Previous reports have shown that overexpression of resistance-nodulation-cell division (RND)-type efflux pumps is associated with tigecycline resistance in Enterobacteriaceae, such as AcrAB (Ruzin et al., 2005; Keeney et al., 2007; Bratu et al., 2009; He et al., 2015). Ribosomal protein mutation (via the *rpsJ* gene) has also been reported to cause tigecycline resistance in Enterobacteriaceae (Beabout et al., 2015; He et al., 2018; Xu et al., 2020). Recently, the plasmid-mediated mobile tigecycline resistance gene *tet*(X4) and its variants has been reported in Enterobacteriaceae (He et al., 2019; Sun et al., 2019). However, this gene has most often been reported in *Escherichia coli* strains (Zhang et al., 2020), and its role in *K. pneumoniae* is limited.

In 2018, we reported the first case of tigecycline resistance in CRKP mediated by *tet*(A) evolution *in vivo* during tigecycline treatment (Du et al., 2018). Previously, Linkevicius et al. (2016) observed that evolutionary changes in *tet*(A) can cause tigecycline resistance in *E. coli in vitro*. Chiu et al. (2017) considered widespread mutated *tet*(A) gene to be concerning for the possible dissemination of tigecycline resistance in *K. pneumoniae*. To explore the role of *tet*(A) in tigecycline resistance in clinical CRKP isolates, tigecycline minimum inhibitory concentration (MIC) distribution, *tet*(A)-bearing rate, *tet*(A) mutation rate and transmission ability of clinical CRKP isolates were analyzed through antimicrobial susceptibility tests, whole genome sequencing, bioinformatics analysis, and plasmid conjugation experiments.

## Materials and Methods

### CRKP Clinical Isolates

A total of 63 non-repetitive CRKP clinical strains were continuously collected from April 1st to May 30th in 2018 at a tertiary hospital in Hangzhou, China. Strains from different specimens of the same patient or specimens collected from the same patient at different times were considered to be duplicate strains, and only the first strain was selected for subsequent research. Supplementary Figure 1 outlined the detailed specimen collection information. All of the isolates were identified using the VITEK MS system (bioMérieux, Marcy-l’Étoile, France). The carbapenem resistance genes, *bla*_*KPC*_ and *bla*_*NDM*_, as well as the tetracycline resistance gene, *tet*(A), were amplified by PCR and further sent for Sanger sequencing.

### Antimicrobial Susceptibility Test

Antimicrobial susceptibility testing was conducted using standard broth microdilution tests and the VITEK 2 system (bioMérieux) with Gram-negative antimicrobial susceptibility testing cards (AST-GN13) following the guidelines of the Clinical and Laboratory Standards Institute (CLSI). Antimicrobial agents: amoxicillin/clavulanate, ceftriaxone, cefepime, cefoxitin, aztreonam, piperacillin/tazobactam, imipenem, meropenem, amikacin, levofloxacin, sulfamethoxazole/trimethoprim, colistin, and tigecycline were used in the test. Antimicrobial susceptibility was determined using breakpoints approved by the CLSI (2019). For tigecycline MIC detection, standard broth microdilution tests were adopted with fresh (<12 h) Mueller-Hinton broth (Cation-adjusted, Oxoid Ltd., Basingstoke, Hampshire, United Kingdom). *E. coli* ATCC 25922 was used for quality control. As there are no CLSI breakpoints for tigecycline, the FDA standard was adopted^1^. The interpretation of colistin MIC was follow by the EUCAST guideline (Breakpoints for 2021)^2^.

### Quantitative Real-Time PCR

mRNA expression levels of the efflux pump genes, *acrA* and *acrB*, in tigecycline-resistant isolates were examined by quantitative real-time PCR according to our previously published paper (He et al., 2015). The relative expression of each target gene was calibrated against the corresponding expression of *K. pneumoniae* type strain ATCC 13883 (expression = 1), which served as a control with a tigecycline MIC of 0.125 mg/L. Relative expression levels of the two genes were analyzed by the 2^–Δ^ ^Δ^ ^*CT*^ analytical method.

### Whole-Genome Sequencing

Isolates confirmed to possess the *tet*(A) gene or resistance to tigecycline were sent for whole-genome sequencing using the Illumina NovaSeq 6000 platform (Illumina Inc., San Diego, CA, United States). In brief, genomic DNA was extracted using a QIAamp DNA Mini Kit (Qiagen, Valencia, CA, United States) and sent for sequencing using the paired-end 2 × 150-bp protocol. The draft genome sequences were assembled using SPAdes 3.13.0. Three strains with *tet*(A) mutants (CRKP52R, CRKP66R, and CRKP78R) were further sent for Nanopore sequencing with a long-read MinION sequencer (Nanopore, Oxford, United Kingdom). Both short Illumina reads and long MinION reads were hybrid assembled using Unicycler (v0.4.7). Complete genome sequences were generated and automatically annotated by the NCBI Prokaryotic Genome Annotation Pipeline (PGAP) server.

### Genomic and Phylogenetic Relationship Analysis of *tet*(A)-Positive Isolates

MLST, acquired antibiotic resistance genes (ARGs) and plasmid replicons were analyzed using the BacWGSTdb 2.0 server (Ruan and Feng, 2016; Feng et al., 2020; Ruan et al., 2020). The phylogenetic relationship between *tet*(A)-carrying isolates was analyzed using the (neighbor joining (NJ))/unweighted pair group method with arithmetic mean (UPGMA) phylogeny method (MAFFT version 7) based on a core genome single nucleotide polymorphism strategy (Katoh et al., 2019). A phylogenetic tree was constructed using the resulting SNPs with recombination regions removed using the maximum parsimony algorithm (Jia et al., 2019). The KL type of *K. pneumoniae* was predicted by Kaptive Web (Wick et al., 2018).

### Tigecycline Resistance Evolution Experiment *in vitro*

Wild-type *tet*(A)-carrying CRKP clinical isolates were used as parental strains in tigecycline resistance evolution experiments *in vitro*. Tigecycline-resistant mutants were selected by successive passages in MH broth containing increasing concentrations of tigecycline. In brief, one single clone of the parental strain was inoculated in MH broth overnight, and 200 μL of overnight cultures was added to 2 mL of fresh MH broth containing serial concentrations of tigecycline. The selective concentration began at 0.5 mg/L and doubled every 24 h. The protocol was repeated until the mutants grew at a tigecycline concentration of 32 mg/L.

### Conjugation Experiment and VITEK MS Identification

Tigecycline-resistant *tet*(A) mutants obtained from tigecycline resistance evolution experiments and their parental strains with wild-type *tet*(A) were used as donors, and rifampicin-resistant *E. coli* EC600 was used as the recipient. Transconjugants were selected on MH agar plates supplemented with tetracycline (16 mg/L) and rifampicin (600 mg/L). *E. coli* EC600 transconjugants were identified using the VITEK MS system, and *tet*(A) gene mutations were further confirmed by PCR and Sanger sequencing. The conjugation efficiency was measured and calculated following the protocol in https://openwetware.org/wiki/conjugation.

### Characterization of the *tet*(A)-Bearing Plasmid and Genetic Background of *tet*(A)

Circular comparisons of the *tet*(A)-carrying plasmid were conducted with BLAST Ring Image Generator (BRIG) based on concentric rings (Alikhan et al., 2011). Insertion elements (ISs) located on the plasmids were predicted by application of ISfinder (Siguier et al., 2006). Integrative and conjugative elements (ICEs) were predicted using ICEberg (Liu et al., 2019). The genetic location and background of *tet*(A) were determined by aligning the contigs carrying *tet*(A) with complete genome sequences generated in this study using CLC Genomics Workbench 10.0.1.

### Nucleotide Sequence Accession Numbers

We deposited the complete sequences of the CRKP52R, CRKP66R, and CRKP78R *K. pneumoniae* strains and plasmids in GenBank under accession numbers CP066249-CP066253, CP063833-CP063838, and CP066254-CP066259. The draft genome sequences of 33 *tet*(A)-positive strains were deposited in GenBank under accession numbers JAEQKY000000000-JAEQME000000000. The sequence with GenBank accession number AJ517790 was used as the reference for the wild-type *tet*(A) gene with the primary start codon of GTG.

## Results

### Tigecycline MIC Distribution and Mutations in *rpsJ*, *ramR*, and *tet*(A)

**FIGURE 1:**
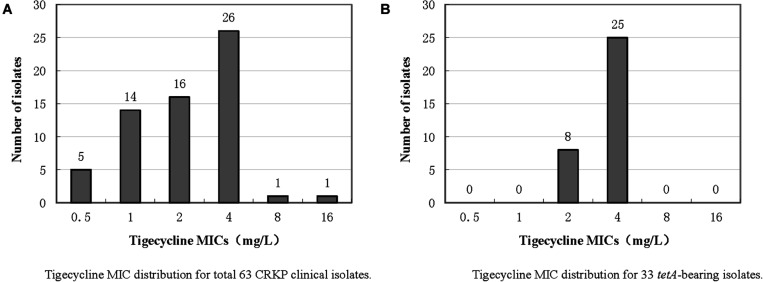
Tigecycline MIC distributions of 63 CRKP clinical isolates and 33 *tet*(A)-bearing isolates.

The antimicrobial susceptibility testing results are presented in Supplementary Table 1. All isolates were multidrug resistant bacteria with a resistance rate greater than 85% compared to β-lactams, quinolones and aminoglycosides but were still highly sensitive to colistin. The tigecycline MIC distribution of the 63 isolates is presented in Figure 1A. All 63 isolates carried *bla*_*KPC–*__2_ gene, and the *in silico* MLST analysis showed that all strains, except three ST437 strains, one ST751 strain, and one ST15 strain, belonged to ST11. The highest MIC was 16 mg/L for isolate CRKP65, and further WGS results showed that this isolate did not harbor the *tet*(A) gene but had a *rpsJ* mutation (V57L). One isolate, namely, CRKP26, had a tigecycline MIC of 8 mg/L. Further WGS results showed that this strain had a *ramR* mutation (GATCCTG insertion at 222–223 resulted in frameshift mutation) and high expression of the RND efflux pump, AcrAB (Table 1), but did not harbor the *tet*(A) gene. Twenty-six isolates had a MIC of 4 mg/L, and among these, 25 isolates harbored wild-type *tet*(A). According to the FDA standard (MIC >2 mg/L for tigecycline non-susceptible), there were 28 tigecycline non-susceptible isolates, and the non-susceptible rate was 44.4%. The expression levels of the RND efflux pump genes, *acrA* and *acrB*, as well as the mutations in *rpsJ*, *ramR*, and *tet*(A) in tigecycline non-susceptible isolates are presented in Table 1. Four isolates (CRKP5, CRKP15, CRKP21, and CRKP26) had a high expression (two-fold increase compare to reference strain) of the AcrAB efflux pump.

**TABLE 1:** Expression levels of the *acrA* and *acrB* efflux pump genes and mutation of *rpsJ*, *ramR*, and *tet*(A) in tigecycline non-susceptible isolates.

**Isolate**	**MIC (mg/L)^b^**	**Relative expression^a^**		***Tet*(A)^*c*^**	**Mutation**	
		***acrA***	***acrB***		***rpsJ***	***ramR***
ATCC 13883	0.125	1	1	ND	–	–
CRKP5	4	2.16 ± 0.10	1.66 ± 0.31	WT	–	–
CRKP8	4	1.18 ± 0.15	1.27 ± 0.03	WT	–	–
CRKP10	4	1.28 ± 0.11	1.08 ± 0.23	WT	–	–
CRKP15	4	2.38 ± 0.39	2.46 ± 0.40	WT	–	–
CRKP17	4	1.44 ± 0.28	0.99 ± 0.12	WT	–	–
CRKP21	4	2.18 ± 0.15	2.07 ± 0.45	WT	–	–
CRKP22	4	1.03 ± 0.05	0.81 ± 0.17	WT	–	–
CRKP24	4	0.89 ± 0.15	0.75 ± 0.05	WT	–	–
CRKP26	8	3.78 ± 0.29	3.56 ± 0.24	ND	–	GATCCTG insertion at 222–223
CRKP29	4	1.99 ± 0.27	1.83 ± 0.12	WT	–	–
CRKP31	4	0.89 ± 0.20	0.80 ± 0.16	ND		–
CRKP34	4	1.94 ± 0.06	1.81 ± 0.24	WT	–	–
CRKP38	4	1.67 ± 0.30	1.00 ± 0.22	WT	–	–
CRKP39	4	1.42 ± 0.23	0.71 ± 0.06	WT	–	–
CRKP41	4	1.31 ± 0.34	0.78 ± 0.06	WT	–	–
CRKP42	4	0.87 ± 0.12	0.89 ± 0.12	WT	–	–
CRKP43	4	0.78 ± 0.06	0.67 ± 0.02	WT	–	–
CRKP45	4	1.20 ± 0.09	0.98 ± 0.12	WT	–	–
CRKP51	4	1.41 ± 0.28	1.14 ± 0.10	WT	–	–
CRKP52	4	1.35 ± 0.15	1.13 ± 0.14	WT	–	–
CRKP55	4	1.79 ± 0.28	1.25 ± 0.15	WT	–	–
CRKP59	4	1.59 ± 0.60	1.17 ± 0.19	WT	–	–
CRKP61	4	0.81 ± 0.14	0.77 ± 0.15	WT	–	–
CRKP62	4	1.02 ± 0.13	0.73 ± 0.06	WT	–	–
CRKP65	16	1.30 ± 0.25	1.14 ± 0.03	ND	G169C (V57L)	–
CRKP72	4	1.03 ± 0.15	1.28 ± 0.28	WT	–	–
CRKP77	4	1.91 ± 0.54	1.32 ± 0.33	WT	–	–
CRKP80	4	1.17 ± 0.34	0.92 ± 0.12	WT	–	–

*^*a*^Relative expression compared with *K. pneumoniae* type strain ATCC 13883 (expression = 1). Results represent the means of three runs ± standard deviation.*

*^*b*^Tigecycline MIC.*

*^*c*^ND, *tet*(A) not detected. WT, wild-type *tet*(A).*

### Characterizations of *tet*(A)-Positive Isolates and Phylogenetic Analysis

**FIGURE 2:**
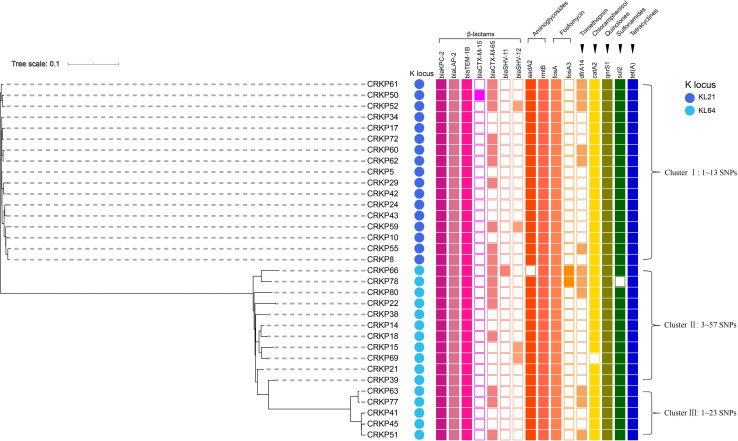
Phylogenetic relationship, antimicrobial resistance genes and serotype analysis based on WGS data of 33 *tet*(A)-bearing CRKP isolates. The cells in different colors indicate the presence of the antimicrobial resistance gene, while the blank cells indicate the absence of the gene. The color of each circle indicates a distinct capsular serotype.

The PCR and Sanger sequencing results showed that 33 of the 63 isolates (52.4%) carried the *tet*(A) gene, all of which were wild-type. The tigecycline MIC distribution of the 33 *tet*(A)-positive isolates is presented in Figure 1B, and there were 25 isolates with MIC 4 mg/L (75.8%) and eight isolates (24.2%) with MIC 2 mg/L. Compared with *tet*(A)-negative isolates, the tigecycline MIC is generally increased by two-fold. All 33 isolates belonged to ST11. The antimicrobial resistance genes and serotype based on WGS data of these 33 *tet*(A)-bearing isolates are presented in Figure 2. In total, 16 antimicrobial resistance genes were found in these isolates, including *bla*_*KPC–*__2_, *bla*_*LAP–*__2_, *bla*_*TEM–*__1__*B*_, *fosA*, *qnrS1*, *rmtB*, *tet*(A), *bla*_*CTX–M–*__15_, *bla*_*CTX–M–*__65_, *bla*_*SHV–*__11_, *bla*_*SHV–*__12_, *aadA2*, *dfrA14*, *catA2*, *fosA3*, and *sul2*. These isolates were divided into two serotypes (KL21 and KL64). The phylogenetic tree is presented in Figure 2, and SNP differences are presented in Supplementary Figure 2. Three clonal clusters (cluster I, cluster II, and cluster III) were identified. All 17 isolates belonged to serotype KL21 (cluster I), which differed with 13 SNPs. According to the relatedness criteria recommended for SNP typing schemes of *K. pneumoniae* reported by Schürch et al. (2018), a difference of SNPs ≤18 represents epidemiologically related. Therefore, a clonal spread of *tet*(A)-positive ST11 *K. pneumoniae* with serotype KL21 occurred in the sampling hospital.

### Mutations of *tet*(A) in the Tigecycline Resistance Evolution Experiment

All 33 wild-type *tet*(A)-carrying CRKP isolates were subjected to tigecycline resistance evolution experiments *in vitro*. Under successive passages of tigecycline induction in MH broth for approximately 2 weeks, 28 isolates were induced to develop resistance with a tigecycline MIC ≥32 mg/L. PCR and Sanger sequencing detected 20 of the 28 isolates that evolved *tet*(A) mutations (Table 2), and the mutation rate was 71.4%. The following eight amino acid substitutions were identified in these mutants: A264V, I248L, A370V, S251A, G300E, G300V, A53G, and G237V. The most common amino acid substitution was A370V, which appeared six times, followed by S251A and G300E (appeared four times each). All 20 *tet*(A) mutants were subjected to conjugation experiments in which they were used as donors, and *E. coli* EC600 was the recipient. Twelve mutants succeeded in the conjugation experiment, and the conjugation efficiency was at a frequency of 10^–3^–10^–8^ (Table 2). The MICs of tigecycline and tetracycline of *E. coli* EC600 transconjugants of *tet*(A) mutants and wild-type *tet*(A) are presented in Table 2. In general, tigecycline MICs in *E. coli* EC600 transconjugants of mutated *tet*(A) were 2- to 8-fold higher than *E. coli* EC600 transconjugants of wild-type *tet*(A), and the MICs of tetracycline also increased in eight strains.

**TABLE 2:** Tigecycline and tetracycline MICs of *E. coli* EC600 transconjugants of *tet*(A) mutants or wild-type *tet*(A).

**Isolates**	**TGC^c^ MICs (mg/L)**	***tet*(A)^a^**	**TGC MICs after tigecycline induction (mg/L)**	***tet*(A) mutation after tigecycline induction^b^**		**Conjugation efficiency**	***E. coli* EC600 transconjugant of *tet*(A) mutant^c^**		***E. coli* EC600 transconjugant of wild-type *tet*(A)^c^**	
				**Nucleotide change**	**Amino acid change**		**TGC (mg/L)**	**TC (mg/L)**	**TGC (mg/L)**	**TC (mg/L)**
CRKP5	4	WT	64	–	–	–	–	–	–	–
CRKP8	4	WT	64	C791T	A264V	(1.4 ± 0.6) × 10^–4^	1	64	0.25	64
CRKP10	4	WT	64	–	–	–	–	–	–	–
CRKP14	2	WT	64	A742C	I248L	(4.8 ± 1.9) × 10^–6^	2	>256	0.5	128
CRKP15	4	WT	64	–	–	–	–	–	–	–
CRKP17	4	WT	64	–	–	–	–	–	–	–
CRKP18	2	WT	64	–	–	–	–	–	–	–
CRKP21	4	WT	64	–	–	–	–	–	–	–
CRKP22	4	WT	4	–	–	–	–	–	–	–
CRKP24	4	WT	64	C1109T	A370V	Failed	NA	NA	NA	NA
CRKP29	4	WT	64	T751G	S251A	(6.0 ± 3.4) × 10^–6^	1	256	0.25	128
CRKP34	4	WT	4	–	–	–	–	–	–	–
CRKP38	4	WT	64	T751G	S251A	(1.8 ± 0.9) × 10^–3^	1	64	0.25	64
CRKP39	4	WT	64	G899T	G300V	Failed	NA	NA	NA	NA
CRKP41	4	WT	4	–	–	–	–	–	–	–
CRKP42	4	WT	64	C1109T	A370V	Failed	NA	NA	NA	NA
CRKP43	4	WT	64	G710T T751G	G237V S251A	Failed	NA	NA	NA	NA
CRKP45	4	WT	4	–	–	–	–	–	–	–
CRKP50	2	WT	64	C791T	A264V	(7.7 ± 3.3) × 10^–7^	1	128	0.5	64
CRKP51	4	WT	64	G899A	G300E	(3.7 ± 1.9) × 10^–6^	2	>256	0.25	128
CRKP52	4	WT	64	G899A	G300E	(5.2 ± 2.5) × 10^–8^	1	256	0.25	128
CRKP55	4	WT	64	C1109T	A370V	(3.2 ± 1.4) × 10^–5^	1	128	0.5	128
CRKP59	4	WT	64	C1109T	A370V	(1.1 ± 0.7) × 10^–5^	1	>256	0.25	128
CRKP60	2	WT	64	G899A	G300E	Failed	NA	NA	NA	NA
CRKP61	4	WT	64	–	–	–	–	–	–	–
CRKP62	4	WT	4	–	–	–	–	–	–	–
CRKP63	2	WT	32	–	–	–	–	–	–	–
CRKP66	2	WT	64	C1109T	A370V	(1.4 ± 1.2) × 10^–6^	1	64	0.25	64
CRKP69	2	WT	64	G899A	G300E	(1.1 ± 0.5) × 10^–4^	4	>256	0.5	128
CRKP72	4	WT	64	C158G	A53G	Failed	NA	NA	NA	NA
CRKP77	4	WT	64	C1109T	A370V	Failed	NA	NA	NA	NA
CRKP78	2	WT	64	T751G	S251A	(4.7 ± 2.8) × 10^–6^	4	>256	0.5	128
CRKP80	4	WT	64	C791T	A264V	Failed	NA	NA	NA	NA

*^*a*^WT, wild-type *tet*(A).*

*^*b*^–, no mutation detected in *tet*(A).*

*^*c*^TGC, Tigecycline; TC, Tetracycline. NA, Data not available.*

### Characterization of the *tet*(A)-Bearing Plasmid and Genetic Background of *tet*(A)

**FIGURE 3:**
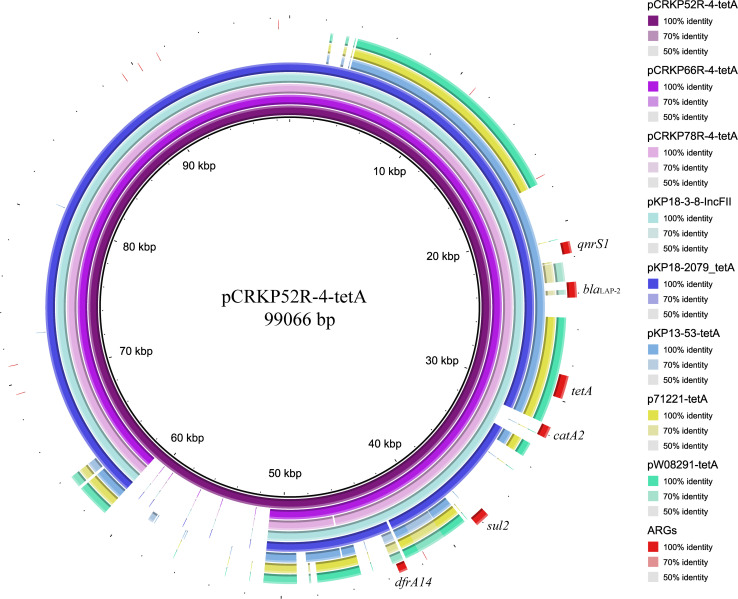
Plasmid backbone comparisons of *tet*(A)-carrying plasmids. Plasmid information is presented in Table 3. ARGs are indicated in red.

**FIGURE 4:**
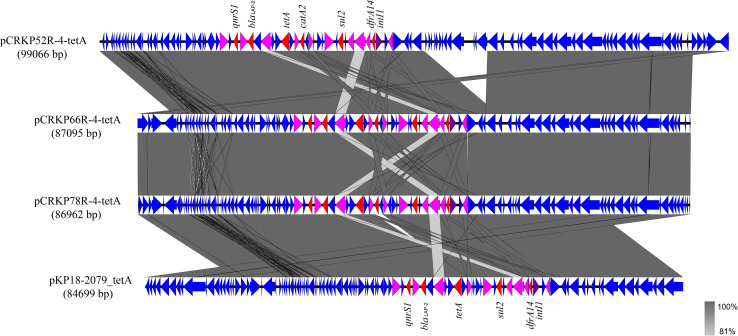
Alignment of integrative elements with *tet*(A) in the pCRKP52R-4-tetA, pCRKP66R-4-tetA, pCRKP78R-4-tetA, and pKP18-2079_tetA plasmids. Resistance genes are indicated in red, and *int1* is indicated in purple. IS elements are indicated in pink, and all other ORFs are indicated in blue.

The complete genome sequences of three *tet*(A) mutants (CRKP52R, CRKP66R, and CRKP78R) were obtained using Nanopore sequencing. CRKP52R, CRKP66R, and CRKP78R were tigecycline-resistant *tet*(A) mutants collected in the tigecycline resistance evolution experiment. The parental strains of CRKP52R, CRKP66R, and CRKP78R were *K. pneumoniae* strains CRKP52, CRKP66, and CRKP78, respectively. *tet*(A) was located on plasmids in these strains, and three *tet*(A)-bearing plasmids were identified in CRKP52R, CRKP66R, and CRKP78R. The plasmid from CRKP52R was a ColRNAI/IncFII plasmid that was 99,066 bp in size and was designated pCRKP52R-4-tetA. Plasmids from CRKP66R and CRKP78R belonged to the IncFII type with sizes of 87,095 and 86,962 bp, and they were designated pCRKP66R-4-tetA and pCRKP78R-4-tetA, respectively (Table 3). Similar *tet*(A)-bearing plasmids in the NCBI GenBank database were searched with the Basic Local Alignment Search Tool (BLAST). We found that IncFII-type and IncFIB-type plasmids with sizes of approximately 90 and 120 kb were common plasmids carrying *tet*(A) in *K. pneumoniae* (Table 3). The similarity of the plasmid backbone of these plasmids is presented in Figure 3. These IncFII-type plasmids have a plasmid backbone similar to that of ARGs, including *qnrS1*, *bla*_*LAP–*__2_, *tet*(A), *catA2*, *sul2*, and *dfrA14*. The *tet*(A) genes in the pCRKP52R-4-tetA plasmid were flanked by the *qnrS1* and *bla*_*LAP–*__2_ resistance genes on the left and the *catA2*, *sul2*, and *dfrA14* resistance genes on the right, and they were all located in ICEs ranging from 11 to 99 kb in size (Figure 3). One class 1 integron (IntI1) was identified in pCRKP52R-4-tetA (from 18944 to 46168 bp), pCRKP66R-4-tetA, and pCRKP78R-4-tetA (Figure 4). This integron contains multiple ARGs, including *tet*(A). The *tet*(A) gene was located in the genetic environment, IS*26*-*tetR*-*tet*(A)-*eamA*-*orf-*TnAs1, suggesting that it was acquired by horizontal gene transfer with mobilizable transposons.

**TABLE 3:** Detailed information of *tet*(A)-bearing plasmids obtained in this study and the NCBI database.

**Plasmid name**	**Plasmid replicon**	**Plasmid size**	**Host bacteria**	**Antimicrobial resistance genes**	**Accession number**
pCRKP52R-4-tetA	ColRNAI/IncFII	99066bp	*K. pneumoniae*	*qnrS1*, *bla*_*LAP–*__2_, *tet*(A), *catA2*, *sul2*, *dfrA14*	CP066252 (this study)
pCRKP66R-4-tetA	IncFII	87095bp	*K. pneumoniae*	*qnrS1*, *bla*_*LAP–*__2_, *tet*(A), *catA2*, *sul2*, *dfrA14*	CP063836 (this study)
pCRKP78R-4-tetA	IncFII	86962bp	*K. pneumoniae*	*qnrS1*, *bla*_*LAP–*__2_, *tet*(A), *catA2*, *sul2*, *dfrA14*	CP066257 (this study)
pKP18-3-8-IncFII	IncFII	87095bp	*K. pneumoniae*	*qnrS1*, *bla*_*LAP–*__2_, *tet*(A), *catA2*, *sul2*, *dfrA14*	MT035876 (NCBI)
pKP18-2079_tetA	IncFII	84699bp	*K. pneumoniae*	*qnrS1*, *bla*_*LAP–*__2_, *tet*(A), *sul2*, *dfrA14*	MT090960 (NCBI)
pKP13-53-tet(A)	Col/IncFIB	181383bp	*K. pneumoniae*	*qnrS1*, *bla*_*LAP–*__2_, *tet*(A), *sul1*, *dfrA1*, *aac(3)-IId*	MN268580 (NCBI)
p71221-tetA	IncFIB	128170bp	*K. pneumoniae*	*tet*(A), *sul1*, *dfrA1*, *mph(A)*, *bla*_*SHV–*__12_, *aph(3′)-Ia*	MN310374 (NCBI)
pW08291-tetA	IncFIB	130483bp	*K. pneumoniae*	*tet*(A), *sul1*, *dfrA1*, *mph(A)*, *bla*_*SHV–*__12_	MN310376 (NCBI)

## Discussion

Carbapenem-resistant *K. pneumoniae* has emerged as an important pathogen worldwide, and the emergence of tigecycline and colistin resistance makes clinical treatment difficult. According to the data obtained in this study, 52.4% of the tested isolates carried the wild-type *tet*(A) gene. Among these isolates, 75.8% of these *tet*(A)-bearing isolates exhibited a tigecycline non-susceptible phenotype. No mutations of *rpsJ* and *ramR* genes were identified in these isolates. We also searched the plasmid-encoded RND efflux pump genes *tmexCD1-toprJ1* in the genome of these strains, but no related genes were found. Compared with *tet*(A)-negative isolates, the tigecycline MIC was generally increased by approximately two-fold in these wild-type *tet*(A)-bearing isolates. Except for three isolates (CRKP5, CRKP15, and CRKP21) had a high expression of the AcrAB efflux pump (Table 1), we considered that other undiscovered mechanisms may be utilized in these isolates that work together with wild-type *tet*(A) to mediate tigecycline non-susceptibility. A recent study reported that TetA in synergy with RND-type efflux transporters contribute to tigecycline resistance in *Acinetobacter baumannii* (Foong et al., 2020). This synergy may also exist in *K. pneumoniae*, which warrant further investigation.

*tet*(A) is a MFS family efflux pump, and mutation in *tet*(A) might result in increased accumulation of tigecycline as a substrate, thus contributing to tigecycline resistance (Linkevicius et al., 2016; Chiu et al., 2017). *tet*(A)-bearing *K. pneumoniae* tended to more easily evolve tigecycline resistance under selective pressure as 71.4% of the strains evolved *tet*(A) mutations and developed high-level tigecycline resistance in our tigecycline resistance evolution experiment *in vitro*. We have previously confirmed the contribution of the S251A Tet(A) variant to tigecycline resistance by transformation experiments (Du et al., 2018). Linkevicius et al. (2016) also confirmed that *tet*(A) mutants of I235F, I248L, S251A, and G300E show increased tigecycline MICs compared to the unmutagenized control. We further conducted transformation experiments on several other mutants (A264V, A370V, G300V, and A53G) identified in this study, and we found these mutants increased the tigecycline MIC in *E. coli* DH5α by 2 to 4-fold compared to the wild-type *tet*(A) control. The degree of tigecycline MIC increase in *E. coli* EC600 transconjugants of *tet*(A) mutants was diverse (Table 2), especially for a few transconjugants harboring the same mutation site (e.g., CRKP29, CRKP38, and CRKP78). Linkevicius et al. (2016) confirmed that the magnitude increase of tigecycline MIC depends on the expression level of the *tet*(A) mutant. Thus, we speculated that the difference of the *tet*(A) mutant expression level may be due to the diverse tigecycline MICs in these *E. coli* EC600 transconjugants. *tet*(A) mutants are often located in different types of plasmids, and these plasmids have different promoter and regulatory sequences that may result in different expression levels of *tet*(A).

Multiple types of *tet*(A)-bearing plasmids were retrieved from the NCBI GenBank database, and circular comparison analysis revealed that they have some similar structures, suggesting that genetic exchange and recombination among different types of *tet*(A)-bearing plasmids have occurred (Ribera et al., 2003; Szmolka et al., 2015; Yao et al., 2020). In the three *tet*(A)-bearing plasmids obtained in this study, one class 1 integron containing multiple ARGs, including *tet*(A), was detected. In addition, *tet*(A) mutation occurring under selective pressure may lead to tigecycline treatment failure. Zhang et al. (2019) reported the coexistence of *mcr-1* and the *tet*(A) variant on the same plasmid from a *K. pneumoniae* isolate in human gut, and Yao et al. (2020) also reported an IncFII plasmid co-harboring *bla*_*IMP–*__26_ and *tet*(A) variant in a clinical *K. pneumoniae* isolate. It seems that *tet*(A) mutants can not only occur in *bla*_*KPC–*__2_-carrying plasmids, but also form fusion plasmids with other carbapenem resistance genes and *mcr* gene, which will cause a higher transmission risk of simultaneous resistance to carbapenem, colistin and tigecycline. The emergence and spread of such fusion plasmid needs our attention.

## Conclusion

In conclusion, our study revealed the wide-spread of plasmid-borne *tet*(A) gene in clinical CRKP, and mutation of *tet*(A) is a potential threat that lead to tigecycline resistance. More attention should be devoted to monitoring the spread of plasmid-borne *tet*(A) in *K. pneumoniae* clinical isolates, especially the emergence of *tet*(A) mutants. Strict administration of tigecycline and classification management of antibiotics must be carried out with precautions.

## Data Availability Statement

The data presented in the study are deposited in the GenBank repository, accession numbers CP066249-CP066253, CP063833-CP063838, CP066254-CP066259, and JAEQKY000000000-JAEQME000000000.

## Ethics Statement

This study was conducted in accordance with the Declaration of Helsinki and approved by the Ethics Committee of Zhejiang Provincial People’s Hospital. Written informed consent from the patients was exempted by the Ethics Committee of Zhejiang Provincial People’s Hospital because the present study only focused on bacteria.

## Author Contributions

FH designed the experiments. JX and ZZ performed the experiments and were the major contributors in writing the manuscript. YC and WW analyzed the data. All authors read and approved the final manuscript.

## Conflict of Interest

The authors declare that the research was conducted in the absence of any commercial or financial relationships that could be construed as a potential conflict of interest.

## Footnotes

1 https://www.fda.gov/drugs/development-resources/tigecycline-injection-products

2 https://eucast.org/

## Supplementary Material

Supplementary Figure 1 Specimen type and department distribution of 63 non-repetitive CRKP clinical isolates.

Supplementary Figure 2 Phylogenetic analysis of 33 *tet*(A)-bearing isolates based on the core genome SNP strategy.
