# Supplementary figures and images for: The Plasmid-Borne tet(A) Gene Is an Important Factor Causing Tigecycline Resistance in ST11 Carbapenem-Resistant Klebsiella pneumoniae Under Selective Pressure
Source: Front Microbiol. 2021 Feb 24;12:644949. doi: 10.3389/fmicb.2021.644949 (PMC7943888; doi:10.3389/fmicb.2021.644949)

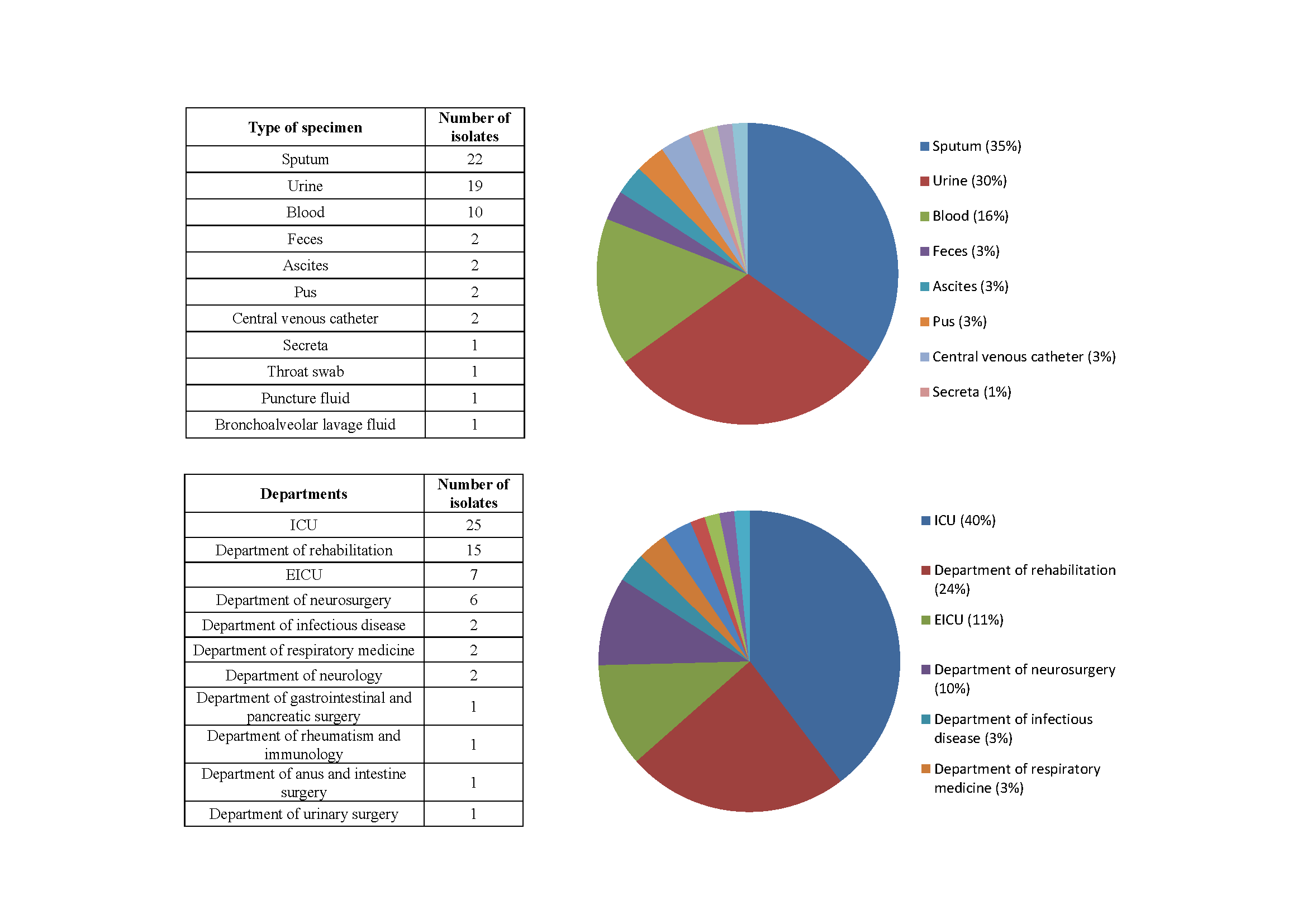

Supplement: Supplementary Figure 1 — Specimen type and department distribution of 63 non-repetitive CRKP clinical isolates. [file Image_1.TIF]
