# Supplementary material for: The Plasmid-Borne tet(A) Gene Is an Important Factor Causing Tigecycline Resistance in ST11 Carbapenem-Resistant Klebsiella pneumoniae Under Selective Pressure
Source: Front Microbiol. 2021 Feb 24;12:644949. doi: 10.3389/fmicb.2021.644949 (PMC7943888; doi:10.3389/fmicb.2021.644949)
Supplement: Supplementary file 3 [file Table_1.DOCX]

Table S1. Susceptibilities of 63 CRKP isolates to 13 antimicrobial agents.

| **Isolates** | MIC (mg/L) ^a^ | | | | | | | | | | | | |
| --- | --- | --- | --- | --- | --- | --- | --- | --- | --- | --- | --- | --- | --- |
|  | AMC^b^ | CRO^b^ | FEP^b^ | FOX^b^ | ATM^b^ | TZP^b^ | IMP^b^ | MEM^c^ | AMK^b^ | LVF^b^ | SXT^b^ | CT^c^ | TGC^c^ |
| CRKP1 | ≥32 | ≥64 | ≥64 | ≥64 | ≥64 | ≥128 | ≥16 | ＞128 | ≥64 | ≥8 | ≤1/19 | 0.0625 | 2 |
| CRKP2 | ≥32 | ≥64 | 8 | ≥64 | ≥64 | ≥128 | ≥16 | 64 | ≤2 | ≥8 | ≤1/19 | 0.0625 | 2 |
| CRKP5 | ≥32 | ≥64 | ≥64 | ≥64 | ≥64 | ≥128 | ≥16 | ＞128 | ≥64 | ≥8 | ≥16/304 | 0.125 | 4 |
| CRKP7 | ≥32 | ≥64 | ≥64 | ≥64 | ≥64 | ≥128 | ≥16 | ＞128 | ≥64 | ≥8 | ≤1/19 | 0.125 | 1 |
| CRKP8 | ≥32 | ≥64 | ≥64 | ≥64 | ≥64 | ≥128 | ≥16 | ＞128 | ≥64 | ≥8 | ≤1/19 | 0.5 | 4 |
| CRKP10 | ≥32 | ≥64 | ≥64 | ≥64 | ≥64 | ≥128 | ≥16 | ＞128 | ≥64 | ≥8 | ≤1/19 | 0.25 | 4 |
| CRKP14 | ≥32 | ≥64 | ≥64 | ≥64 | ≥64 | ≥128 | ≥16 | ＞128 | ≥64 | ≥8 | ≤1/19 | 0.125 | 2 |
| CRKP15 | ≥32 | ≥64 | ≥64 | ≥64 | ≥64 | ≥128 | ≥16 | ＞128 | ≥64 | ≥8 | ≤1/19 | 0.0625 | 4 |
| CRKP17 | ≥32 | ≥64 | ≥64 | ≥64 | ≥64 | ≥128 | ≥16 | ＞128 | ≥64 | ≥8 | ≥16/304 | 0.0625 | 4 |
| CRKP18 | ≥32 | ≥64 | ≥64 | ≥64 | ≥64 | ≥128 | ≥16 | ＞128 | ≥64 | ≥8 | ≤1/19 | 0.125 | 2 |
| CRKP19 | ≥32 | ≥64 | ≥64 | ≥64 | ≥64 | ≥128 | ≥16 | ＞128 | ≥64 | ≥8 | ≥16/304 | 0.0625 | 0.5 |
| CRKP20 | ≥32 | ≥64 | 4 | 16 | ≥64 | ≥128 | ≥16 | 64 | ≤2 | 1 | ≤1/19 | 0.125 | 0.5 |
| CRKP21 | ≥32 | ≥64 | ≥64 | ≥64 | ≥64 | ≥128 | ≥16 | ＞128 | ≥32 | ≥8 | ≤1/19 | 0.0625 | 4 |
| CRKP22 | ≥32 | ≥64 | ≥64 | ≥64 | ≥64 | ≥128 | ≥16 | ＞128 | ≥64 | ≥8 | ≥16/304 | 0.0625 | 4 |
| CRKP24 | ≥32 | ≥64 | ≥64 | ≥64 | ≥64 | ≥128 | ≥16 | ＞128 | ≥64 | ≥8 | ≥16/304 | 0.0625 | 4 |
| CRKP26 | ≥32 | ≥64 | ≥64 | ≥64 | ≥64 | ≥128 | ≥16 | ＞128 | ≥64 | ≥8 | ≤1/19 | 0.0625 | 8 |
| CRKP28 | ≥32 | ≥64 | ≥64 | ≥64 | ≥64 | ≥128 | ≥16 | ＞128 | ≥64 | ≥8 | ≥16/304 | 0.25 | 1 |
| CRKP29 | ≥32 | ≥64 | ≥64 | ≥64 | ≥64 | ≥128 | ≥16 | ＞128 | ≥64 | ≥8 | ≥16/304 | 0.0625 | 4 |
| CRKP30 | ≥32 | ≥64 | ≥64 | ≥64 | ≥64 | ≥128 | ≥16 | ＞128 | ≥64 | ≥8 | ≤1/19 | 0.0625 | 2 |
| CRKP31 | ≥32 | ≥64 | ≥64 | ≥64 | ≥64 | ≥128 | ≥16 | ＞128 | ≥64 | ≥8 | ≤1/19 | 0.0625 | 4 |
| CRKP32 | ≥32 | ≤1 | ≥64 | ≤4 | ≥64 | ≥128 | ≥16 | ＞128 | ≤2 | ≤0.25 | ≤1/19 | 0.0625 | 0.5 |
| CRKP34 | ≥32 | ≥64 | ≥64 | ≥64 | ≥64 | ≥128 | ≥16 | ＞128 | ≥64 | ≥8 | ≥16/304 | 0.0625 | 4 |
| CRKP36 | ≥32 | ≥64 | ≥64 | ≥64 | ≥64 | ≥128 | ≥16 | 32 | ≥64 | ≥8 | ≤1/19 | 0.0625 | 1 |
| CRKP37 | ≥32 | ≥64 | 8 | ≥64 | ≥64 | ≥128 | ≥16 | 32 | ≥64 | ≥8 | ≤1/19 | 0.0625 | 1 |
| CRKP38 | ≥32 | ≥64 | ≥64 | ≥64 | ≥64 | ≥128 | ≥16 | ＞128 | ≥64 | ≥8 | ≥16/304 | 0.0625 | 4 |
| CRKP39 | ≥32 | ≥64 | ≥64 | ≥64 | ≥64 | ≥128 | ≥16 | ＞128 | ≥64 | ≥8 | ≥16/304 | 0.0625 | 4 |
| CRKP40 | ≥32 | ≥64 | ≥64 | ≥64 | ≥64 | ≥128 | ≥16 | ＞128 | ≥64 | ≥8 | ≤1/19 | 0.0625 | 2 |
| CRKP41 | ≥32 | ≥64 | ≥64 | ≥64 | ≥64 | ≥128 | ≥16 | ＞128 | ≥64 | ≥8 | ≥16/304 | 0.0625 | 4 |
| CRKP42 | ≥32 | ≥64 | ≥64 | ≥64 | ≥64 | ≥128 | ≥16 | ＞128 | ≥64 | ≥8 | ≥16/304 | 0.0625 | 4 |
| CRKP43 | ≥32 | ≥64 | ≥64 | ≥64 | ≥64 | ≥128 | ≥16 | ＞128 | ≥64 | ≥8 | ≥16/304 | 0.125 | 4 |
| CRKP45 | ≥32 | ≥64 | ≥64 | ≥64 | ≥64 | ≥128 | ≥16 | ＞128 | ≥64 | ≥8 | ≥16/304 | 0.0625 | 4 |
| CRKP46 | ≥32 | ≥64 | 2 | 16 | ≥64 | ≥128 | ≥16 | 64 | ≤2 | ≥8 | ≤1/19 | 0.0625 | 0.5 |
| CRKP47 | ≥32 | ≥64 | 8 | ≥64 | ≥64 | ≥128 | ≥16 | 64 | ≥64 | ≥8 | ≤1/19 | 0.0625 | 1 |
| CRKP48 | ≥32 | ≥64 | ≥64 | ≥64 | ≥64 | ≥128 | ≥16 | ＞128 | ≥64 | ≥8 | ≥16/304 | 0.125 | 1 |
| CRKP50 | ≥32 | ≥64 | ≥64 | ≥64 | ≥64 | ≥128 | ≥16 | ＞128 | ≥64 | ≥8 | ≥16/304 | 0.0625 | 2 |
| CRKP51 | ≥32 | ≥64 | ≥64 | ≥64 | ≥64 | ≥128 | ≥16 | ＞128 | ≥64 | ≥8 | ≥16/304 | 0.0625 | 4 |
| CRKP52 | ≥32 | ≥64 | ≥64 | ≥64 | ≥64 | ≥128 | ≥16 | ＞128 | ≥64 | ≥8 | ≥16/304 | 0.125 | 4 |
| CRKP53 | ≥32 | ≥64 | ≥64 | ≥64 | ≥64 | ≥128 | ≥16 | ＞128 | ≤2 | ≥8 | ≥16/304 | 2 | 1 |
| CRKP54 | ≥32 | ≥64 | ≥64 | ≥64 | ≥64 | ≥128 | ≥16 | 64 | ≥64 | ≥8 | ≤1/19 | 0.0625 | 1 |
| CRKP55 | ≥32 | ≥64 | ≥64 | ≥64 | ≥64 | ≥128 | ≥16 | ＞128 | ≥64 | ≥8 | ≥16/304 | 0.0625 | 4 |
| CRKP56 | ≥32 | ≥64 | 8 | ≥64 | ≥64 | ≥128 | ≥16 | 32 | ≥64 | ≥8 | ≤1/19 | 0.0625 | 1 |
| CRKP57 | ≥32 | ≥64 | ≥64 | ≥64 | ≥64 | ≥128 | ≥16 | ＞128 | ≥64 | ≥8 | ≥16/304 | 0.0625 | 1 |
| CRKP59 | ≥32 | ≥64 | ≥64 | ≥64 | ≥64 | ≥128 | ≥16 | ＞128 | ≥64 | ≥8 | ≤1/19 | 0.125 | 4 |
| CRKP60 | ≥32 | ≥64 | ≥64 | ≥64 | ≥64 | ≥128 | ≥16 | ＞128 | ≥64 | ≥8 | ≥16/304 | 0.125 | 2 |
| CRKP61 | ≥32 | ≥64 | ≥64 | ≥64 | ≥64 | ≥128 | ≥16 | ＞128 | ≥64 | ≥8 | ≥16/304 | 0.0625 | 4 |
| CRKP62 | ≥32 | ≥64 | ≥64 | ≥64 | ≥64 | ≥128 | ≥16 | ＞128 | ≥64 | ≥8 | ≥16/304 | 0.0625 | 4 |
| CRKP63 | ≥32 | ≥64 | ≥64 | ≥64 | ≥64 | ≥128 | ≥16 | ＞128 | ≥64 | ≥8 | ≥16/304 | 0.0625 | 2 |
| CRKP65 | ≥32 | ≥64 | ≥64 | ≥64 | ≥64 | ≥128 | ≥16 | ＞128 | ≥64 | ≥8 | ≤1/19 | ＞64 | 16 |
| CRKP66 | ≥32 | ≥64 | ≥64 | ≥64 | ≥64 | ≥128 | ≥16 | ＞128 | ≥64 | ≥8 | ≥16/304 | 0.125 | 2 |
| CRKP67 | ≥32 | ≥64 | ≥64 | ≥64 | ≥64 | ≥128 | ≥16 | ＞128 | ≥64 | ≥8 | ≥16/304 | 0.0625 | 2 |
| CRKP69 | ≥32 | ≥64 | ≥64 | ≥64 | ≥64 | ≥128 | ≥16 | ＞128 | ≥64 | ≥8 | ≤1/19 | 0.0625 | 2 |
| CRKP70 | ≥32 | ≥64 | ≥64 | ≥64 | ≥64 | ≥128 | ≥16 | 64 | ≤2 | ≥8 | ≥16/304 | 0.125 | 1 |
| CRKP72 | ≥32 | ≥64 | ≥64 | ≥64 | ≥64 | ≥128 | ≥16 | ＞128 | ≥64 | ≥8 | ≤2/38 | 0.125 | 4 |
| CRKP76 | ≥32 | ≥64 | ≥64 | ≥64 | ≥64 | ≥128 | ≥16 | ＞128 | ≥64 | ≥8 | ≥16/304 | 0.0625 | 1 |
| CRKP77 | ≥32 | ≥64 | ≥64 | ≥64 | ≥64 | ≥128 | ≥16 | ＞128 | ≥64 | ≥8 | ≥16/304 | 0.0625 | 4 |
| CRKP78 | ≥32 | ≥64 | ≥64 | ≥64 | ≥64 | ≥128 | ≥16 | ＞128 | ≥64 | ≥8 | ≥16/304 | 0.0625 | 2 |
| CRKP79 | ≥32 | ≥64 | ≥64 | ≥64 | ≥64 | ≥128 | ≥16 | 64 | ≥64 | ≥8 | ≤1/19 | 0.5 | 1 |
| CRKP80 | ≥32 | ≥64 | ≥64 | ≥64 | ≥64 | ≥128 | ≥16 | ＞128 | ≥64 | ≥8 | ≥16/304 | 0.0625 | 4 |
| CRKP81 | ≥32 | ≥64 | ≥64 | ≥64 | ≥64 | ≥128 | ≥16 | 128 | ≥64 | ≥8 | ≥16/304 | 0.25 | 1 |
| CRKP82 | ≥32 | ≥64 | ≥64 | ≥64 | ≥64 | ≥128 | ≥16 | 128 | 8 | ≥8 | 2/38 | 0.0625 | 1 |
| CRKP85 | ≥32 | ≥64 | ≥64 | ≥64 | ≥64 | ≥128 | ≥16 | ＞128 | ≥64 | ≥8 | ≤1/19 | 0.0625 | 1 |
| CRKP87 | ≥32 | ≥64 | 2 | 16 | ≥64 | ≥128 | ≥16 | ＞128 | ≤2 | ≥8 | ≤1/19 | 0.0625 | 1 |
| CRKP88 | ≥32 | ≥64 | 2 | 16 | ≥64 | ≥128 | ≥16 | 32 | ≤2 | ≥8 | ≤1/19 | 0.0625 | 2 |

^a^ Abbreviations: AMC, amoxicillin/clavulanate; CRO, ceftriaxone; FEP, cefepime; FOX, cefoxitin; ATM, aztreonam; TZP, piperacillin/tazobatam; IPM, imipenem; MEM, meropenem; AMK, amikacin; LVF, levofloxacin; SXT, sulfamethoxazole/trimethoprim; CT, colistin; TGC, tigecycline.

^b^ AST-GN13.

^c^ Standard broth microdilution tests.
